# MW-MADDPG: a meta-learning based decision-making method for collaborative UAV swarm

**DOI:** 10.3389/fnbot.2023.1243174

**Published:** 2023-09-21

**Authors:** Minrui Zhao, Gang Wang, Qiang Fu, Xiangke Guo, Yu Chen, Tengda Li, XiangYu Liu

**Affiliations:** ^1^College of Air and Missile Defense, Air Force Engineering University, Xi'an, China; ^2^Graduate School, Academy of Military Science, Beijing, China; ^3^Unit 95866 of PLA, Baoding, China

**Keywords:** UAV, meta learning, multi-agent reinforcement learning (MARL), Model Agnostic Meta Learning (MAML), MADDPG

## Abstract

Unmanned Aerial Vehicles (UAVs) have gained popularity due to their low lifecycle cost and minimal human risk, resulting in their widespread use in recent years. In the UAV swarm cooperative decision domain, multi-agent deep reinforcement learning has significant potential. However, current approaches are challenged by the multivariate mission environment and mission time constraints. In light of this, the present study proposes a meta-learning based multi-agent deep reinforcement learning approach that provides a viable solution to this problem. This paper presents an improved MAML-based multi-agent deep deterministic policy gradient (MADDPG) algorithm that achieves an unbiased initialization network by automatically assigning weights to meta-learning trajectories. In addition, a Reward-TD prioritized experience replay technique is introduced, which takes into account immediate reward and TD-error to improve the resilience and sample utilization of the algorithm. Experiment results show that the proposed approach effectively accomplishes the task in the new scenario, with significantly improved task success rate, average reward, and robustness compared to existing methods.

## 1. Introduction

**Figure 1:**
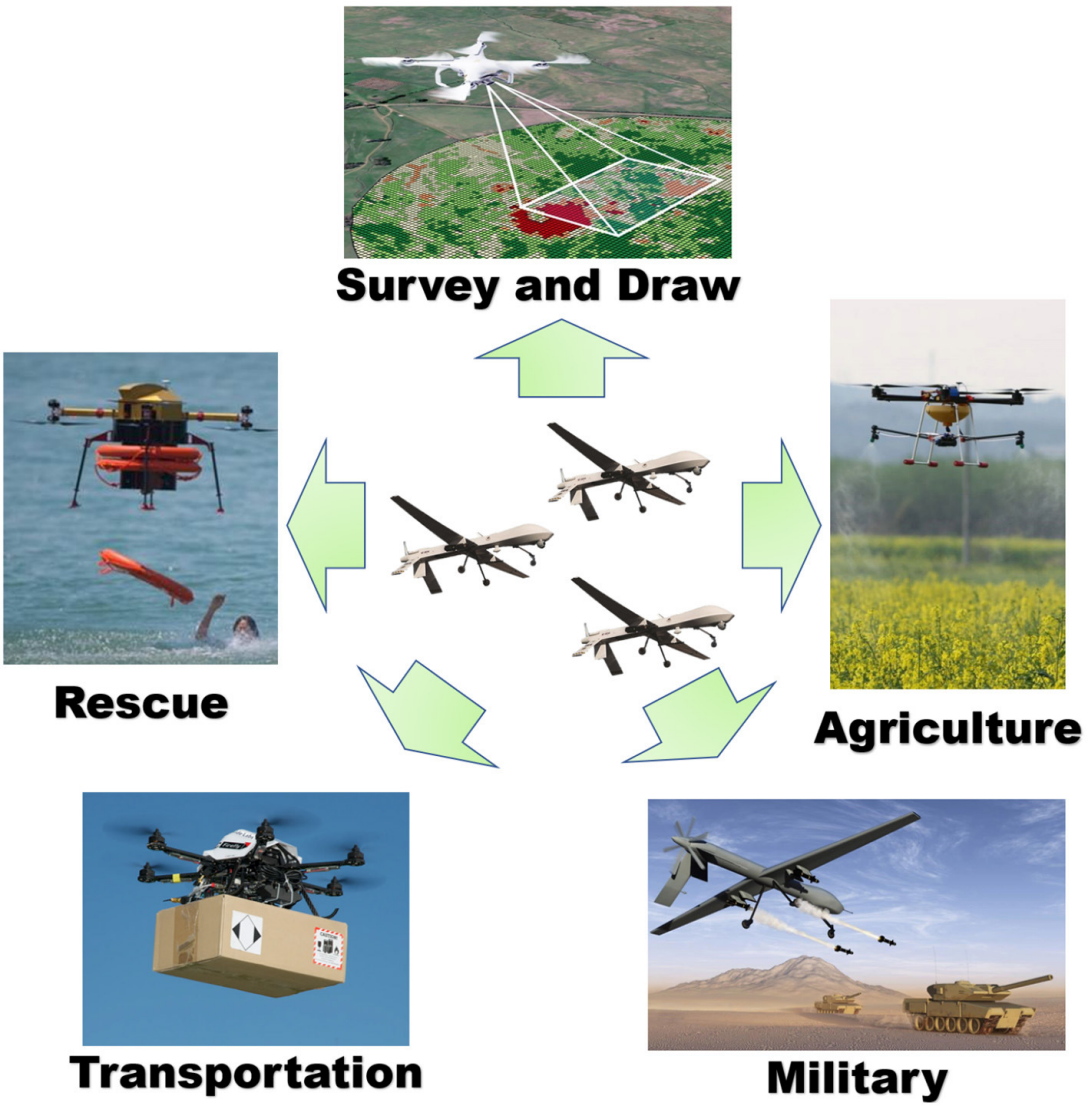
Unmanned aerial vehicles (UAVs) application scope diagram. UAVs have been widely utilized across various fields due to their numerous advantages.

As a reusable vehicle, Unmanned Aerial Vehicles (UAVs) do not need to be piloted. Instead, they are capable of accomplishing the given tasks by remote control or autonomous control (Silveira et al., 2020; Yao et al., 2021). This has received much attention from the industry in recent years. UAVs have several advantages, including low life-cycle cost (Lei et al., 2021), low personnel risk (Rodriguez-Fernandez et al., 2017), long duration of flight (Ge et al., 2022; Pasha et al., 2022), and maneuverability, size, and speed (Poudel and Moh, 2022). These UAVs are increasingly being used in various fields such as tracking targets (Hu et al., 2023), agriculture (Liu et al., 2022b), rescue (Jin et al., 2023), and transportation (Li et al., 2021) for “Dull, Dirty, Dangerous, and Deep” (4D) missions (Aleksander, 2018; Chamola et al., 2021). The applications of UAVs are illustrated in Figure 1. During a mission, UAVs typically operate in swarms to accomplish their objectives. Consequently, the cooperative control and decision-making methods used by UAV swarms have become increasingly critical. Effective collaborative decision-making techniques can enhance the efficiency and effectiveness of mission accomplishment. However, it is important to note that current cooperative decision-making methods, including non-learning methods and traditional heuristics for UAVs, have limited capacity to effectively manage conflicts between multiple aircraft and maintain a balance between adapting to variable mission environments and meeting time constraints. Therefore, this area has received significant attention from researchers seeking to develop more robust and versatile methods for UAV cooperative decision-making.

At present, methods for cooperative control and decision-making of UAV swarms are typically classified into two main categories: top-down and bottom-up (Giles and Giammarco, 2019). Top-down approaches are primarily utilized for centralized collaborative control and decision-making, while bottom-up approaches are mainly applied to distributed collaborative decision-making and control (Wang et al., 2022).

The main advantage of the top-down approach is its ability to decompose complex tasks into smaller, more manageable components. In the context of UAV swarm collaborative decision-making, this approach can be used to break down the task into a task assignment problem, a trajectory planning problem, and a swarm control problem (Tang et al., 2023). For example, Zhang et al. (2022) proposed a method for assigning search and rescue tasks to a combination of helicopters and UAVs. They analyzed the search and rescue level of each point and the hovering endurance of the UAV using principal component analysis and cluster analysis. They then constructed a multi-objective optimization model and solved it using the non-dominated sorting genetic algorithm-II to assign tasks to the UAVs. Liu et al. (2021) utilized the “Divide and Conquer” approach to create a hierarchical task scheduling framework that decomposed the UAV scheduling problem into several subproblems. They proposed a tabu-list-based simulated annealing (SATL) algorithm for task assignment and a variable neighborhood descent (VND) algorithm for generating the scheduling scheme. In another study, Liu et al. (2022a) proposed a particle swarm optimization algorithm for cluster scheduling of UAVs performing remote sensing tasks in emergency scenarios. While centralized decision-making methods have better global reach and simpler structures, their communication and computational costs increase significantly with an increase in the number of UAVs in the swarm. Therefore, there is a need to develop a distributed cooperative decision-making method for UAV swarms.

The bottom-up approach facilitates cooperative decision-making of UAV swarms through the observation, judgment, decision-making, and distributed negotiation of individual UAVs. This approach aligns well with the observe-orient-decide-act (OODA) theory and is particularly suited for distributed decision-making scenarios (Puente-Castro et al., 2022), which are increasingly becoming the future trend (Ouyang et al., 2023).

Wang and Zhang (2022) proposed a UAV cluster task allocation method based on the bionic wolf pack approach, which decomposes task allocation into three processes: task assignment, path planning, and coverage search. The UAV swarm is modeled according to the characteristics of a wolf pack, and distributed collaborative decision-making is achieved through information sharing within the UAV swarm. Yang et al. (2022) presented a distributed task reallocation method for the dynamic environment where tasks need to be reassigned among a UAV swarm. They proposed a distributed decision framework based on time-type processing policies and used a partial reassignment algorithm (PRA) to generate conflict-free solutions with less data communication and faster execution. Wei et al. (2021) introduced a distributed UAV cluster computational offloading method that leverages distributed Q-learning and proposes a cooperative exploration-based, prioritized experience replay method using distributed deep reinforcement learning techniques. This approach achieves distributed computational offloading and outperforms traditional methods in terms of average processing time, energy-task efficiency, and convergence rate (Ouyang et al., 2023).

In recent years, deep reinforcement learning has shown promising results in various fields, such as training championship-level racers in Gran Turismo (Wurman et al., 2022), achieving all-time top-three Stratego game ranking (Perolat et al., 2022), and optimizing matrix multiplication operations (Fawzi et al., 2022). However, when addressing the challenge of cooperative decision-making in UAV swarms, reinforcement learning suffers from weak generalization ability, low sample utilization, and slow learning speed (Beck et al., 2023). To address these challenges, researchers have turned to meta-reinforcement learning, which is currently a hot topic in machine learning.

Meta-learning, also referred to as learn to learn, is a technique that involves training on a relevant task to learn meta-knowledge, which can then be applied to a new environment. This approach reduces the number of samples required and increases the training speed in the new environment (Hospedales et al., 2022). Researchers have proposed meta-reinforcement learning methods by combining meta-learning with reinforcement learning techniques. Meta-reinforcement learning enhances the generalization ability and learning efficiency by utilizing the acquired meta-knowledge to guide the subsequent training process and achieve cross-task learning with limited samples (Beck et al., 2023). Despite its successful implementation in various fields (Chen et al., 2022; Jiang et al., 2022; Zhao et al., 2023), meta-reinforcement learning has not yet been widely adopted in the field of cooperative decision-making for heterogeneous UAV swarms.

The experience replay mechanism is a critical technique in deep reinforcement learning, first proposed in the deep Q network model (Mnih et al., 2015). It improves data utilization, increases policy stability, and breaks correlations between states in the training data. To measure the priority of experience, Hou et al. (2017) proposed a method that uses the Temporal-Difference (TD) error, which improves the convergence speed of the algorithm. Pan et al. (2022) proposed a TD-Error and Time-based experience sampling method to reduce the influence of outdated experience. Li et al. (2022) introduced a Clustering experience replay (CER) method that clusters and replays transition using a divide-and-conquer framework based on time division, effectively exploiting the experience hidden in all explored transitions in the current training. However, prioritized experience replay algorithms that only consider TD-error in the learning process tend to ignore the role of immediate payoffs and experience with small time-differential errors, and the learning effectiveness of the algorithm is susceptible to the detrimental effects of temporal error outliers.

In this paper, we propose an improved MAML-based MADDPG algorithm to enhance the generalization capability, learning rate, and robustness of deep reinforcement learning methods used in UAV swarm collaborative decision-making for heterogeneous UAV swarms. The proposed algorithm incorporates a Reward-TD prioritized experience replay mechanism and buffer experience forgetting mechanism to improve the overall performance of the system. Firstly, the paper describes the problem of cooperative attack on ground targets by UAV swarms, models the UAV motion model, and formulates the cooperative decision-making problem as a POMDP model. Next, inspired by the Meta Weight Learning algorithm (Xu et al., 2021), the paper proposes an improved meta-weight multi-agent deep deterministic policy gradient (MW-MADDPG) algorithm to obtain an unbiased initialization model by setting playback weights for trajectories and updates the meta-weights by gradient and momentum. To increase the effectiveness of the experience replay mechanism, the paper proposes a Reward-TD prioritized experience replay method with a forgetting mechanism. Finally, experiments are conducted to verify the generalization, robustness, and learning rate of the proposed approach. The main contributions of this paper include:

1. Proposing the meta-weight multi-agent deep deterministic policy gradient (MW-MADDPG) algorithm for UAV swarm collaborative decision-making, which achieves end-to-end learning across tasks and can be applied to new scenarios quickly and stably after training.
2. Introducing the Reward-TD prioritized experience replay method to improve the convergence speed and utilization of experiences in the MW-MADDPG algorithm. The proposed method determines the priority of experience replay based on immediate reward and TD-error, thereby enhancing the quality of experience replay.
3. Employing a forgetting mechanism in the proposed MW-MADDPG algorithm to improve algorithm robustness and reduce overfitting. A threshold of sampling times is set to reduce the repetition of a small number of experiences during the experience replay process.

## 2. Background

### 2.1. Reinforcement learning

Reinforcement learning is a trial-and-error technique for continuous learning, where an agent interacts with its external environment. The objective of the agent is to obtain the maximum cumulative reward from the external environment. Typically, reinforcement learning models the problem as a Markov decision process (MDP) or a partially observable Markov decision process (POMDP), which allows the agent to make decisions based on current states and future rewards, without requiring knowledge of the full environment model. Through repeated interactions with the environment, the agent learns through experience to select actions that lead to higher cumulative rewards, thereby improving its performance over time. A Markov reward process is usually represented by the tuple *M* = < *S, A, T, R*, γ >, where: *S* = (*s*_1_, *s*_2_, ⋯   , *s*_n_), *S* is the set of all possible states in the MDP; *A* = (*a*_1_, *a*_2_, ⋯   , *a*_m_), *A* denotes the set of all possible actions in the MDP, γ ∈ [0, 1], is the discount factor, which indicates the degree of influence of future rewards on the current behavior of the agents. γ = 1 indicates that the future reward has the same effect as the current reward. γ = 0 indicates that the future reward does not affect the current intelligence's action. In the reinforcement learning process, at each time step t, the intelligence is in state *s*_*t*_, observes the environment, takes action *a*_*t*_, gets feedback from the environment *R*_*t*_, and moves to the next state *s*_*t*+1_. In an MDP, a state is called a Markov state when it satisfies the following conditions:


(1) $$\begin{array}{l} P\left[s_{t+1}|s_{t}\right]=P\left[s_{t+1}|s_{1},\cdots \;,s_{t},\right] \end{array}$$

The property that the state of the next moment is independent of the state of the past moment is known as the Markov property. In a Markov decision process (MDP), the state transition matrix *P* (also known as the state transition probability matrix) specifies the probability of transitioning from the current state *s* to the subsequent state *s*′. Specifically, each element $P_{ss^{\prime }}$ represents the probability of transitioning from state *s* to state *s*′ under a given action.


(2) $$\begin{array}{l} P_{ss^{\prime }}=P\left[s_{t+1}=s^{\prime }|s_{t}=s\right] \end{array}$$

The reward *R*_*t*_ is also called cumulative reward, which is the sum of all rewards from the beginning to the end of the round:


(3) $$\begin{array}{l} R_{t}=\overset{\infty }{\underset{k=0}{\sum }}\gamma^{k}r_{t+k+1} \end{array}$$

The reward function indicates that the agent takes action *a*, and the expected reward after the transfer:


(4) $$\begin{array}{l} r_{s}^{a}=𝔼\left[r_{t+1}|s_{t}=s,a_{t}=a\right] \end{array}$$

### 2.2. Multi-agent reinforcement learning

In a multi-agent system, each agent has a limited observation range and can only obtain local information, making it challenging to observe the global environment. This problem is modeled as a Decentralized Partially Observable Markov Decision Process (Dec-POMDP) defined by the tuple *M* = < *N, S, A, P, R, O*, γ >. Here, *N* represents the set of agents, *S* represents the set of agent states, *A* = *A*_1_ × *A*_2_ × ⋯ × *A*_*N*_ represents the joint action set of agents, where the action set of agent *i* is *A*_*i*_, with *i* ∈ [1, *N*]. The state transition function *P*:*S* × *A* × *S* → [0, 1] represents the probability of equipment transition. R is the reward function for all agents, and *O* = *O*_1_ × *O*_2_ × ⋯ × *O*_*N*_ represents the joint observation value of agents, where *O*_*i*_ denotes the observation value of agent *i*. Finally, γ ∈ [0, 1] is the discount factor.

In Dec-POMDP, all agents select actions based on their own observations *O*_*i*_ in the state *s*_*t*_, leading to a transition to the next state *s*_*t*+1_ and receiving an environmental reward value *r*_*i*_. The goal of each agent is to maximize the cumulative reward $G=\overset{T}{\underset{t=0}{\sum }}\gamma^{t}r_{i}^{t}$. This paper employs the classical MARL algorithm MADDPG, with further details provided in Section 4.1.

### 2.3. Meta-learning

**Figure 2:**
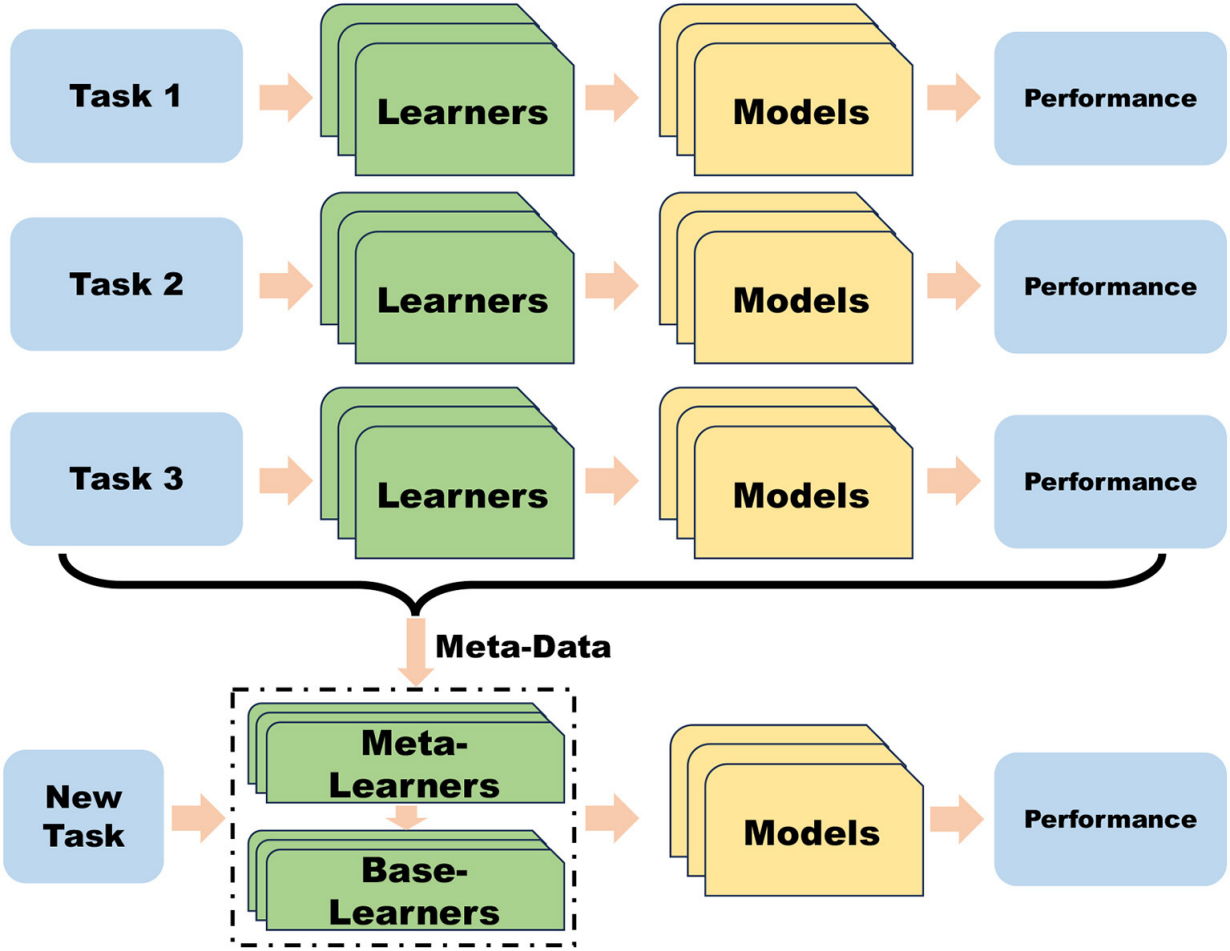
Schematic diagram of the meta-learning process. Meta-learning facilitates rapid adaptation to new tasks by leveraging knowledge acquired from previous tasks.

Meta-learning, also known as learn-to-learn, is a recent research direction aimed at training an initial model to quickly adapt to new tasks with fewer data. Meta-learning comprises three phases: meta-training, meta-validation, and meta-testing. In the meta-training phase, a neural network uses support set data to train for a set of tasks and learn general knowledge for these tasks. In the meta-validation phase, the neural network selects query set data to verify model generalization and adjust hyperparameters used in meta-learning. Finally, in the meta-testing stage, the model is tested on new tasks to evaluate its training effect. The meta-learning paradigm is depicted in Figure 2. The formal definition of meta-reinforcement learning is presented below, whereas the learning task of reinforcement learning is:


(5) $$\begin{array}{l} T=\left\{L_{T},P_{T}\left(s\right),P_{T}\left(s_{t+1}|s_{t},a_{t}\right),H\right\} \end{array}$$

Here, *L*_*T*_ represents the loss function that maps a given trajectory τ = (*s*_0_, *a*_1_, *s*_1_, *r*_1_, …, *a*_*H*_, *s*_*H*_, *r*_*H*_) to a loss value. *P*_*T*_(*s*) denotes the initial state distribution, while *P*_*T*_(*s*_*t*+1_|*s*_*t*_, *a*_*t*_) refers to the state transition probability distribution. *H* corresponds to the trajectory length.

This paper discusses Model Agnostic Meta Learning (MAML), which is a model-independent general meta-learning algorithm that can be applied to any algorithm trained using gradient descent. MAML is adapted to deep neural network models through the use of meta-gradient updates and can be used for various neural network architectures such as convolutional, fully connected, recurrent neural networks, and more. Additionally, it can be applied to different types of machine-learning problems, such as regression, classification, clustering, reinforcement learning, and others.

The main idea of Model-Agnostic Meta-Learning (MAML) is to obtain an initial model that can be applied to a range of tasks and requires only a small amount of task-specific training to achieve good performance. Specifically, the strategy π_θ_ is obtained by interacting with the environment through the strategy π_θ_, collecting *K* trajectories $\tau_{\theta }^{1:K}$, with the goal of minimizing the loss on the new task distribution *D*(*T*) and obtaining the strategy π_ϕ_.

MAML updates the parameters ϕ of the strategy π_ϕ_ by computing the gradient of the loss function $L_{T}\left(\tau_{\theta }^{1:K}\right)$ w.r.t. the parameter θ, and updating ϕ as:


(6) $$\begin{array}{l} \phi =\theta -\beta \nabla_{\theta }L_{T}\left(\tau_{\theta }^{1:K}\right) \end{array}$$

Here, $L_{T}\left(\tau_{\theta }^{1:K}\right)$ is the average loss over *K* trajectories, where $\tau_{\theta }^{k}\sim P_{T}\left(\tau |\theta \right)$. The loss function *L*_*T*_(τ_θ_) for each trajectory τ_θ_ is defined as:


(7) $$\begin{array}{l} L_{T}\left(\tau_{\theta }\right)=-{𝔼}_{s_{t},a_{t\sim \pi_{\theta }},P_{T}\left(s\right)}\left[\overset{H}{\underset{t=1}{\sum }}r\left(s_{t},a_{t}\right)\right] \end{array}$$

where β is the meta-learning rate.

## 3. Problem formulation

### 3.1. Task description

**Figure 3:**
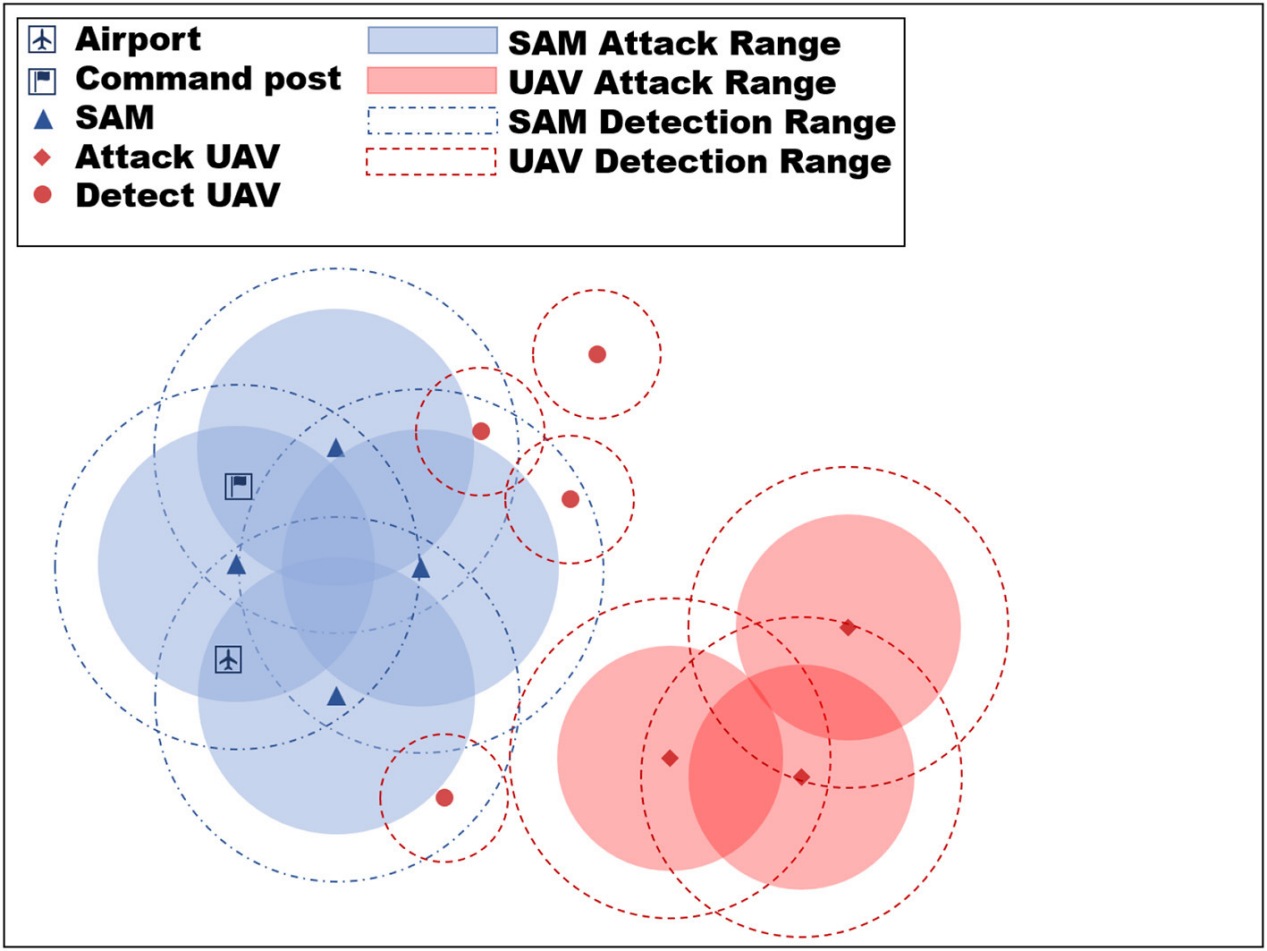
Experimental environment diagram. The objective of the red UAV swarm is to eliminate the blue airports and command posts, while the blue SAM is tasked with defending these targets.

The objective of the UAV in the paper is to destroy the opponent's (blue side) strategic key location and ensure the survival of our side as much as possible while achieving this objective. The blue's strategic location is protected by Surface-to-air missiles (SAMs), which have a longer detection and attack range than our UAVs. Thus, it is imperative for the Red UAVs to exhibit cooperative behavior to successfully achieve the mission objective, which may involve the strategic “sacrifice” of detecting UAVs for locating SAM positions when necessary while minimizing the loss of attack UAVs. The neural network's strategy generation through learning is reliant on the adversary's strategy during training. Typically, the opponent's strategies are formulated by humans, which limits the samples to encompass the entire situation. To circumvent this issue, this work incorporates a large number of random variables into the SAM strategy modeling, such as the randomization of firing timing, firing number, and firing units. These variations introduce a dynamic battlefield environment in each confrontation, posing a challenge for the neural network. Although we know the location of the blue's strategic key location beforehand, we do not know the location of their SAMs, which can vary from mission to mission. Therefore, the red-side UAV algorithm needs to have fast adaptation capability. Figure 3 in the paper shows the experimental environment.

#### 3.1.1. Force setting

Red side:

- Attack UAV: 3, detection range 35 km, attack range 30 km each carrying four anti-radiation missiles (ARM), four air-to-ground missiles (ATG);
- Detect UAV: 4, detection range 10 km.

Blue side:

- Strategic key location: command post, airport;
- SAM: three sets, each set is called a fire unit, attack range 35 km, with a guidance radar detection range of 40 km.

#### 3.1.2. Winning rules

Red side:

- Victory condition: command post is destroyed;
- Failure condition: command post is not destroyed at the endgame.

Blue side:

- Victory condition: command post is not destroyed at the endgame;
- Failure condition: command post is destroyed.

#### 3.1.3. Battlefield environment settings

- The red side is unable to detect the position of the blue side's SAMs until the guidance radar of the blue side's fire unit is activated;
- The information collected by the Red Detect UAV regarding fire units is automatically synchronized and shared with other Red UAVs;
- In each game, the position of the fire unit will remain‘unchanged;
- The guidance radar of the fire units must be activated before they are able to launch their missiles;
- Once the guidance radar of the fire units is turned on, it cannot be turned off again;
- If the guidance radar of the fire unit is destroyed, the fire unit becomes inoperable and unable to launch missiles;
- The guidance radar must be activated during the guidance procedure;
- If the guidance radar of a fire unit is destroyed, any missiles launched by that unit will immediately self-destruct;
- The ARM and ATG have a shooting range of 30 km and an 80% hit rate;
- In the kill zone, ARM, ATG have a high kill probability of 75% and a low kill probability of 55%.

### 3.2. UAV kinematic model

Typically, the flight control of UAVs involves considering their six degrees of freedom, such as heading, pitch, and roll. However, in this paper, we focus on studying the application of deep reinforcement learning methods in multi-UAV cooperative mission planning while taking into account the maneuvering performance of UAVs, which generally do not perform large-angle maneuvers or drastic changes in acceleration. Therefore, we establish a simplified UAV motion model as follows:


(8) $$\begin{array}{l} \left[\begin{array}{c} {ẋ}_{i}\left(t\right)\\ {ẏ}_{i}\left(t\right)\\ {\dot{\varphi }}_{i}\left(t\right)\\ {\dot{v}}_{i}\left(t\right) \end{array}\right]=\left[\begin{array}{c} v_{i}\left(t\right)\cos\varphi_{i}\left(t\right)\\ v_{i}\left(t\right)\sin\varphi_{i}\left(t\right)\\ \varpi_{i}\left(t\right)\\ {ū}_{i}\left(t\right) \end{array}\right] \end{array}$$

where (*x*_*i*_, *y*_*i*_) denotes the position of UAV *i*, φ_*i*_ and*v*_*i*_ denote the heading angle and velocity of UAV *i*, and ϖ_*i*_and ū_*i*_ denote the angular velocity and acceleration of UAV.

The UAV motion model has the following motion constraints:


(9) $$\begin{array}{l} \{\begin{array}{c} 0\leq x_{i}\leq x_{\max}\\ 0\leq y_{i}\leq y_{\max}\\ v_{\min}\leq v_{i}\leq v_{\max}\\ \varphi_{\min}\leq \varphi_{i}\leq \varphi_{\max} \end{array} \end{array}$$

### 3.3. POMDP model

This section models the decision problem for the UAVs as a POMDP and defines the observation space, action space, and reward function.

#### 3.3.1. Observation space

In this paper, the state space for the UAV decision-making process includes the necessary information for the UAVs. For UAV *i*, the observation space is defined as *O*_*i*_ = (*x*_*i*_, *y*_*i*_, φ*i, v*_*i*_, *c*_*ij*_, *o*_*ik*_). Here, $c_{ij}=\left(x_{j},y_{j},\varphi_{j},v_{j},a_{j}^{t-1}\right)$ represents the information obtained by UAV *i* from UAV *j* within its observation range. The action of UAV *j* at the previous moment is denoted by $a_{j}^{t-1}=\left(\varpi_{j}\left(t-1\right),{ū}_{j}\left(t-1\right),M_{j}\left(t-1\right)\right)$, where *M*_*j*_(*t* − 1) represents the action taken by UAV *j* in firing a missile. Additionally, $o_{ik}=\left(x_{k},y_{k},R_{k}^{t-1},M_{k}^{t-1}\right)$ represents the information of fire unit *k* within UAV *i*'s observation range. Here, $R_{k}^{t-1}$ denotes the state of the radar of fire unit *k* at the previous moment, while $M_{k}^{t-1}$ denotes the last moment of missile-firing action taken by fire unit *k*.

Let the set of all UAVs be defined as *D* = {*UAV*_1_, …, *UAV*_*i*_…, *UAV*_*n*_}. Here, *UAV*_*i*_ represents the UAV numbered *i* and *n* is the total number of UAVs. Similarly, let the set of all fire units be defined as *F* = {*F*_1_, …, *F*_*k*_…, *F*_*h*_}, where *F*_*k*_ denotes the fire unit numbered *k*, and *h* is the total number of fire units.

#### 3.3.2. Action space

The action space in this paper includes angular velocity, acceleration, launch missile, and radar state. The specific action space is defined as shown in Table 1.

**Table 1:** Actions definition.

**Action variable**	**Description**
ϖ_*i*_(*t*)	Angular velocity of UAV *i* at moment *t*
ū_*i*_(*t*)	Acceleration of UAV *i* at moment *t*
*M*_*i*_(*t*)	The target number of missile attacks fired by UAV/launch unit *i* at time *t*, which has an initial value of 0
$R_{k}^{t}$	Fire unit *k* radar state at moment *t* (0 for off, 1 for on)

#### 3.3.3. Reward function

The reward design should account for a large number of units on both the blue and red sides, resulting in a significant amount of status and action space. Providing a single reward value at the end of each battle round may result in sparse rewards and make it difficult for agents to explore winning states independently. Therefore, it is essential to create a well-designed reward function that can guide the agent's learning process effectively.

The approach is to assign a reward value for each type of unit on both the red and blue sides, such that the loss or victory of a unit during the battle triggers an appropriate bonus value (negative for losses suffered by our side, positive for those suffered by the opposing side). Additionally, to encourage the UAV to approach the fire unit, a reward is provided when the UAV moves closer to the target.

Providing rewards solely based on wins and losses can result in long training times and sparse rewards, particularly due to the duration of each round. To expedite the training process and enhance the quality of feedback provided during training, additional reward types such as episodic rewards, key event-driven rewards, and distance-based rewards are incorporated. The detailed reward design is presented in Table 2.

**Table 2:** UAV reward definition.

**Categories**	**Event name**	**Weights**	**Description**
Episodic	Win	10	Win
Reward	Loss	0	Loss
Event	Destroyed command post	5	UAV destroys opponent's command post
Based	Destroy airport	3	UAV destroy opponent's airport
Reward	Destroy fire unit radar	2	Destroy a fire unit Radar
	Detect UAV destroyed	−0.5	One of detect UAV was destroyed
	Attack UAV destroyed	−1	One of attack UAV was destroyed
Distance based reward		λ·*d*_*i*_	$d_{i}=\min(\sqrt{{\left(x_{i}-x_{k}\right)}^{2}+{\left(y_{i}-y_{k}\right)}^{2}}$, *i* ∈ *D, k* ∈ *F*) Weighting factor λ determines the magnitude of the distance-based reward

## 4. Method

### 4.1. MADDPG-based collaborative decision-making method

Traditional single-agent reinforcement learning algorithms face challenges when dealing with collaborative multi-UAV tasks, such as large action spaces and unstable environments. In a multi-agent system, the increase in the number of agents leads to a larger state and action space. In addition, each agent's actions dynamically affect the environment in a way that does not exist in a static environment. For these reasons, traditional single-agent reinforcement learning algorithms are ineffective in a multi-agent environment. To address this problem, this paper employs the MADDPG algorithm in the framework of centralized training and decentralized execution. This approach alleviates the difficulties associated with fully centralized or fully decentralized algorithms by striking a balance between the two.

In contrast to traditional DRL algorithms, the MADDPG algorithm can leverage global information during training while utilizing only local information for decision-making. The following method is employed:

Suppose there are M agents in the multi-agent system, with a set of strategy networks denoted as μ = (μ_1_, μ_2_, ⋯   , μ_*M*_), where μ_*i*_ represents the strategy network of the i-th agent. Additionally, there is a set of value networks denoted as *q* = (*q*_1_, *q*_2_, ⋯   , *q*_*M*_), where *q*_*i*_ represents the value network of the i-th agent. The parameter set for the strategy network is denoted as θ = (θ_1_, θ_2_, ⋯   , θ_*M*_), where θ_*i*_ represents the strategy parameters of the *i*-th agent. Similarly, the parameter set for the value network is denoted as ω = (ω_1_, ω_2_.⋯   , ω_*M*_), where ω_*i*_ represents the value network parameters of the *i*-th agent. The objective function for the i-th agent is expressed as follows:


(10) $$\begin{array}{l} J^{i}\left(\theta \right)={𝔼}_{S}\left[q\left(S,\left[\mu_{1}\left(O_{1},\theta_{1}\right),\mu_{2}\left(O_{2},\theta_{2}\right),\cdots \;,\mu_{M}\left(O_{M},\theta_{M}\right)\right]\right)\right] \end{array}$$

For the deterministic strategy μ_*i*_, the strategy gradient can be expressed as:


(11) $$\begin{array}{l} \nabla_{\theta_{i}}J\left(\mu_{i}\right)\\ ={𝔼}_{S}\left[\nabla_{\theta_{i}}q\left(S,\left[\mu_{1}\left(O_{1},\theta_{1}\right),\mu_{2}\left(O_{2},\theta_{2}\right),\cdots \;,\mu_{M}\left(O_{M},\theta_{M}\right)\right]\right);\omega_{i}\right] \end{array}$$

Here, ∇ represents the gradient operator.

A state is sampled from the experience pool D as follows: $s_{t}=\left(o_{t}^{1},\cdots \;,o_{t}^{M}\right)$, which can be used as an observation of the random variable. The agent's action is obtained from the policy network as:


(12) $$\begin{array}{l} a_{t}^{1}=\mu \left(o_{t}^{1};\theta_{1}\right),\cdots \;,a_{t}^{M}=\mu \left(o_{t}^{M};\theta_{M}\right) \end{array}$$

The gradient of the objective function is:


(13) $$\begin{array}{l} g_{\theta }^{i}=\nabla_{\theta_{i}}\mu_{i}\left(o_{t}^{i};\theta_{i}\right)\cdot \nabla_{a_{i}}q\left(s_{t},\left[a_{t}^{1},\cdots \;,a_{t}^{M}\right];\omega_{i}\right) \end{array}$$

The updated formula for the policy network parameters is:


(14) $$\begin{array}{l} \theta_{i}\leftarrow \theta_{i}+\beta_{1}g_{\theta }^{i} \end{array}$$

Here, α_1_ represents the Actor learning rate.

The value network is updated through the TD algorithm as follows:

For the value network *q*_*i*_(*s, a*; ω_*i*_) of agent *i*, given the tuple (*s*_*t*_, *a*_*t*_, *r*_*t*_, *s*_*t*+1_), the computational action according to the policy network is given by:


(15) $$\begin{array}{l} a_{t+1}^{1}=\mu \left(o_{t+1}^{1};\theta_{1}\right),\cdots \;,a_{t+1}^{M}=\mu \left(o_{t+1}^{M};\theta_{M}\right) \end{array}$$

Let $a_{t+1}=\left[a_{t+1}^{1},\cdots \;,a_{t+1}^{M}\right]$. The TD target is computed as:


(16) $$\begin{array}{l} y_{t}^{i}=r_{t}^{i}+\gamma q\left(s_{t+1},a_{t+1};\omega_{i}\right) \end{array}$$

The TD-error is calculated as:


(17) $$\begin{array}{l} \delta_{t}^{i}=q_{i}\left(s_{t},a_{t};\omega_{i}\right)-y_{t}^{i} \end{array}$$

The value network parameters are then updated using gradient descent w.r.t. ω_*i*_.

Update target network parameters for each agent *i*:


(18) $$\begin{array}{l} \theta_{i}^{{\;}^{\prime }}\leftarrow \tau_{1}\theta_{i}+\left(1-\tau_{1}\right)\theta_{i}^{{\;}^{\prime }} \end{array}$$

Here, τ_1_ is the soft update parameter.

### 4.2. Improved algorithm for MAML

This paper presents an improvement to the traditional MAML algorithm. The original MAML algorithm employs an average update method during gradient updates for each task in the task distribution. However, this can lead to biased models that perform better on one task than others. To overcome this issue, we propose an improved MAML method that introduces weights during the gradient update of different trajectories and incorporates an automatic weight calculation method. This approach aims to obtain an unbiased initialized network model.

The traditional MAML method updates the gradients of different trajectories without any distinction during the trajectory update process. This paper proposes a trajectory weighting method that leverages the concept of Adam's algorithm and utilizes gradient and momentum values to set the weights. This approach addresses the issue of subjective weight assignment and accelerates the convergence of the objective function to its minimum value.

The objective function for meta-learning in this paper is expressed as:


(19) $$\begin{array}{l} L_{T}\left(\tau_{\xi }^{1:K}\right):=\overset{K}{\underset{k=1}{\sum }}W_{k}L_{T}\left(\tau_{\xi }^{k}\right),\tau_{\xi }^{k}\sim P_{T}\left(\tau |\xi \right) \end{array}$$

Here, to satisfy the normalization condition, let $W_{k}=\frac{w_{k}}{w_{1}+w_{2}+\cdots +w_{K}}$ be the weight of the k-th trajectory, where K is the total number of trajectories.

To obtain the optimal weights $w_{k}^{*}$ that minimize the objective function, we update the weights *w*_*k*_ by computing their gradient. The gradient of the objective function w.r.t. the weights *w*_*k*_ is given as:


(20) $$\begin{array}{l} \begin{array}{c} g_{k}^{t}=\frac{\partial L_{T}\left(\tau_{\xi }^{1:K}\right)}{\partial w_{k}}=\frac{\partial \overset{K}{\underset{k=1}{\sum }}W_{k}L_{T}\left(\tau_{\xi }^{k}\right)}{\partial w_{k}}=\frac{\partial \overset{K}{\underset{k=1}{\sum }}\frac{w_{k}}{w_{1}+w_{2}\ldots +w_{K}}L_{T}\left(\tau_{\xi }^{k}\right)}{\partial w_{k}}\\ =\overset{K}{\underset{i=1}{\sum }}\frac{w_{i}}{{\left(w_{1}+w_{2}\ldots +w_{K}\right)}^{2}}L_{T}\left(\tau_{\xi }^{k}\right)-\overset{K}{\underset{i=1}{\sum }}\frac{w_{i}L_{T}\left(\tau_{\xi }^{i}\right)}{{\left(w_{1}+w_{2}\ldots +w_{K}\right)}^{2}}\\ =\overset{K}{\underset{i=1}{\sum }}\frac{w_{i}\left[L_{T}\left(\tau_{\xi }^{k}\right)-L_{T}\left(\tau_{\xi }^{i}\right)\right]}{{\left(w_{1}+w_{2}\ldots +w_{K}\right)}^{2}} \end{array} \end{array}$$

Drawing inspiration from the Adam optimization algorithm, we set the following parameters:

First-order momentum: $m_{k}^{t}=\beta_{1}m_{k}^{t-1}+\left(1-\beta_{1}\right)g_{k}^{t}$

Second order momentum: $v_{k}^{t}=\beta_{2}v_{k}^{t-1}+\left(1-\beta_{2}\right){\left(g_{k}^{t}\right)}^{2}$

Bias-corrected first moment estimate:${\hat{m}}_{k}^{t}=m_{k}^{t}/\left(1-\beta_{1}\right)$

Bias-corrected second moment estimate:${\hat{v}}_{k}^{t}=v_{k}^{t}/\left(1-\beta_{2}\right)$

The updated weight for the next time: $w_{k}^{t+1}\leftarrow w_{k}^{t}-\alpha \cdot m_{k}^{t}/\left(\sqrt{{\hat{v}}_{k}^{t}}+\varepsilon \right)$

where β_1_ and β_2_ are exponential decay rates for the moment estimates, ε = 10^−8^ is fuzz factor, α is weight learning rate.

Meta update: $\xi \leftarrow \xi -\beta \nabla_{\xi }\overset{K}{\underset{k=1}{\sum }}W_{k}L_{T}\left(\tau_{\xi }^{k}\right)$

where β is the meta-learning rate.

The proposed improved MAML algorithm is presented in Algorithm 1.

**Algorithm 1:** MW-MADDPG algorithm.

**Input**: Weight learning rate α, meta-learning rate β, and exponential decay rate β_1_, β_2_;
**Input**: The distribution over tasks *P*_*T*_(*s*);
1: Initialize model parameters ξ
2: **for** *i* = 1, ⋯ , *N* **do**
3: Sample batch of tasks *T*_*i*_ ~ *P*_*T*_(*s*)
4: **for** *k* = 1, ⋯ , *K* **do**
5: Sample trajectory $\tau_{\xi }^{k}$ from *T*_*i*_ using Algorithm 2
6: Compute the gradient of $L_{T}\left(\tau_{\xi }^{k}\right)$ w.r.t. ξ_*k*_: $\nabla_{\xi_{k}}L_{T}\left(\tau_{\xi }^{k}\right)$
7: Optimize ξ with gradient descent: $\xi_{i}^{{\;}^{\prime }}=\xi_{i}-\alpha \nabla_{\xi_{k}}L_{T}\left(\tau_{\xi }^{k}\right)$
8: Re-sample K trajectories $\tau_{\xi^{{\;}^{\prime }}}^{1:K}$
9: **end for**
10: **for** all $\tau_{\xi^{{\;}^{\prime }}}^{1:K}$ **do**
11: The objective function w.r.t. the weights *w*_*k*_: $g_{k}^{t}$
12: Compute the first-order and second-order momentum:
$m_{k}=\beta_{1}m_{k-1}+\left(1-\beta_{1}\right)g_{k}^{t}$
$v_{k}=\beta_{2}v_{k-1}+\left(1-\beta_{2}\right){\left(g_{k}^{t}\right)}^{2}$
13: Compute the bias-corrected first and second-moment estimates:
${\hat{m}}_{k}^{t}=m_{k}^{t}/\left(1-\beta_{1}\right)$
${\hat{v}}_{k}^{t}=v_{k}^{t}/\left(1-\beta_{2}\right)$
14: Update the model weights: $w_{k}^{t+1}\leftarrow w_{k}^{t}-\alpha \cdot m_{k}^{t}/\left(\sqrt{{\hat{v}}_{k}^{t}}+\varepsilon \right)$
15: Calculate $W_{k}=\frac{w_{k}}{\overset{K}{\underset{k=1}{\sum }}w_{k}}$ for each trajectory
16: **end for**
17: Meta update: $\xi \leftarrow \xi -\beta \nabla_{\xi }\overset{K}{\underset{k=1}{\sum }}W_{k}L_{T}\left(\tau_{\xi }^{k}\right)$
18: **end for**

### 4.3. Improved prioritized experience replay mechanism

#### 4.3.1. Prioritized experience replay method based on immediate rewards and TD-error

Experience replay methods typically prioritize replay based on the size of TD-error to enhance neural network convergence speed and experience utilization. In this approach, sampling probability is proportional to the absolute value of TD-error, without considering the quality of the experience in supporting task performance. To address this limitation, this paper proposes an experience replay method based on reward and TD-error that includes immediate rewards from actions during the prioritization process. By considering the immediate reward as well as the TD-error, this improved approach can more accurately prioritize experiences that contribute most effectively to task completion.

The priority of TD-error and immediate reward-based experience replay is defined as:


(21) $$\begin{array}{l} P_{T}\left(i\right)=|\delta_{t}^{i}|+\varepsilon \end{array}$$

where ε is a small constant that ensures the priority value is not zero.

The priority based on immediate rewards is given as:


(22) $$\begin{array}{l} P_{r}\left(i\right)=r_{t}^{i}+\varepsilon \end{array}$$

By sorting and ranking these priorities by size, we obtain *rank*_*r*_(*i*) and *rank*_*T*_(*i*). The combined ranking takes both priorities into account and is computed as:


(23) $$\begin{array}{l} rank_{C}\left(i\right)=\rho rank_{r}\left(i\right)+\left(1-\rho \right)rank_{T}\left(i\right) \end{array}$$

Here, ρ denotes the coefficient of importance of the experience which regulates the relative significance of the two experiences under consideration. When ρ = 0, only the TD-error is considered, while when ρ = 1, only the immediate reward is considered.

The combined priority of an experience is given as:


(24) $$\begin{array}{l} P_{C}\left(i\right)={\left(\frac{1}{rank_{C}\left(i\right)}\right)}^{\eta } \end{array}$$

Here, η is the priority importance parameter that determines the degree of consideration given to priority. When η = 0, we have uniform experience sampling.

The experience sampling probability of an experience is obtained by normalizing its combined priority w.r.t. all experiences in the replay buffer:


(25) $$\begin{array}{l} p_{i}=\frac{P_{C}\left(i\right)}{\sum_{j}P_{C}\left(j\right)} \end{array}$$

This probability is used to sample experiences from the replay buffer during the learning process. Experiences with higher combined priorities are more likely to be sampled.

#### 4.3.2. Forgetting mechanism

The immediate reward and TD-error are used to evaluate the learning value of experiences in the replay buffer, but excessive sampling of high-priority experiences can lead to overfitting. To alleviate this issue, this paper introduces a forgetting mechanism to alleviate overfitting.

The forgetting mechanism introduced in this paper includes setting a sampling threshold ψ. When the number of times an experience has been sampled, denoted as *m*_*i*_, exceeds this threshold, its sampling probability is set to zero. This helps prevent overfitting by reducing the impact of experiences that have been repeatedly sampled.

The updated sampling probability of experience *i* after being processed by the forgetting mechanism is denoted as ${p_{i}}^{\prime }$, and is given by:


(26) $$\begin{array}{l} {p_{i}}^{\prime }=\{\begin{array}{cc} p_{i}, & m_{i}\leq \psi \\ 0, & m_{i}>\psi \end{array} \end{array}$$

Here, if *m*_*i*_ is less than or equal to the sampling threshold ψ, the sampling probability of experience *i* remains unchanged (*p*_*i*_). Otherwise, if *m*_*i*_ is greater than ψ, the sampling probability of experience *i* is set to zero. When the replay buffer reaches capacity, experiences are removed in order of sampling replay priority from smallest to largest, based on the grooming of new experiences. This ensures that new experiences can enter the experience pool and contribute to the learning process.

The MADDPG algorithm with an improved prioritized experience replay mechanism is shown in Algorithm 2.

**Algorithm 2:** MADDPG with improved Prioritized Experience Replay.

**Input**: Act noise $N_{t}$, discount factor γ, constant ε, coefficient of importance ρ, priority importance parameter η, sampling threshold ψ, actor
1: learning rate α_1_, and soft update parameter τ_1_;
2: Initialize strategy networks μ = (μ_1_, μ_2_, ⋯ ,
3: μ_*M*_), value networks *q* = (*q*_1_, *q*_2_, ⋯ , *q*_*M*_) and replay
4: buffer *D*
5: **for** *t* = 1 to max-episode-length **do**
6: Observe initial state *s*_1_
7: **for** agent *i* = 1, ⋯ , *M* **do**
8: choose action $a_{t}^{i}=\mu \left(o_{t}^{i};\theta_{i}\right)+N_{t}$ w.r.t.
9: current policy and exploration
10: **end for**
11: Execute action *a*_*t*_ and observe reward *r*_*t*_ and
12: next state *s*_*t*+1_
13: Add experience (*s*_*t*_, *a*_*t*_, *r*_*t*_, *s*_*t*+1_) to replay buffer *D*
14: Sample a minibatch of *B* experiences from
15: *D* using reward-TD prioritized experience replay method with forgetting mechanism
16: **for** *i* = 1, ⋯ , *M* **do**
17: Compute target $y_{t}^{i}=r_{t}^{i}+\gamma q\left(s_{t+1},a_{t+1};\omega_{i}\right)$
18: Compute TD-error: $\delta_{t}^{i}=q_{i}\left(s_{t},a_{t};\omega_{i}\right)-y_{t}^{i}$
19: Compute priority $P_{T}\left(i\right)=|\delta_{t}^{i}|+\epsilon$
20: Compute priority $P_{r}\left(i\right)=r_{t}^{i}+\epsilon$
21: Compute rank *rank*_*T*_(*i*) and *rank*_*r*_(*i*) based on *P*_*T*_(*i*) and *P*_*r*_(*i*), respectively
22: Compute combined rank *rank*_*C*_(*i*) = ρ*rank*_*r*_(*i*)+
23: (1 − ρ)*rank*_*T*_(*i*)
24: Compute combined priority $P_{C}\left(i\right)={\left(\frac{1}{rank_{C}\left(i\right)}\right)}^{\eta }$
25: Compute sampling probability $p_{i}=\frac{P_{C}\left(i\right)}{\sum_{j}P_{C}\left(j\right)}$
26: **if** *m*_*i*_ > ψ **then**
27: $p_{i}^{{\;}^{\prime }}=0$
28: **else if** *m*_*i*_ ≤ ψ **then**
29: $p_{i}^{{\;}^{\prime }}=p_{i}$
30: **end if**
31: **for** agent *i* = 1, ⋯ .*M* **do**
32: Sample a minibatch of *B* samples from *D* using probabilities *p*_*i*_
33: Compute the gradient $g_{\theta }^{i}$ of the policy network of agent i
34: Update policy network parameters:
35: $\theta_{i}\leftarrow \theta_{i}+\alpha_{1}g_{\theta }^{i}$
36: Update the value network parameters by minimizing the loss w.r.t. TD-error:
$L\left(\omega_{i}\right)=\frac{1}{S}\underset{t}{\sum }{\left(\delta_{t}^{i}\right)}^{2}$
37: **end for**
38: Update target network parameters for each agent *i*: $\theta_{i}^{{\;}^{\prime }}\leftarrow \tau_{1}\theta_{i}+\left(1-\tau_{1}\right)\theta_{i}^{{\;}^{\prime }}$
39: **end for**
40: **end for**

## 5. Experiment

### 5.1. Experiment setup

**Figure 4:**
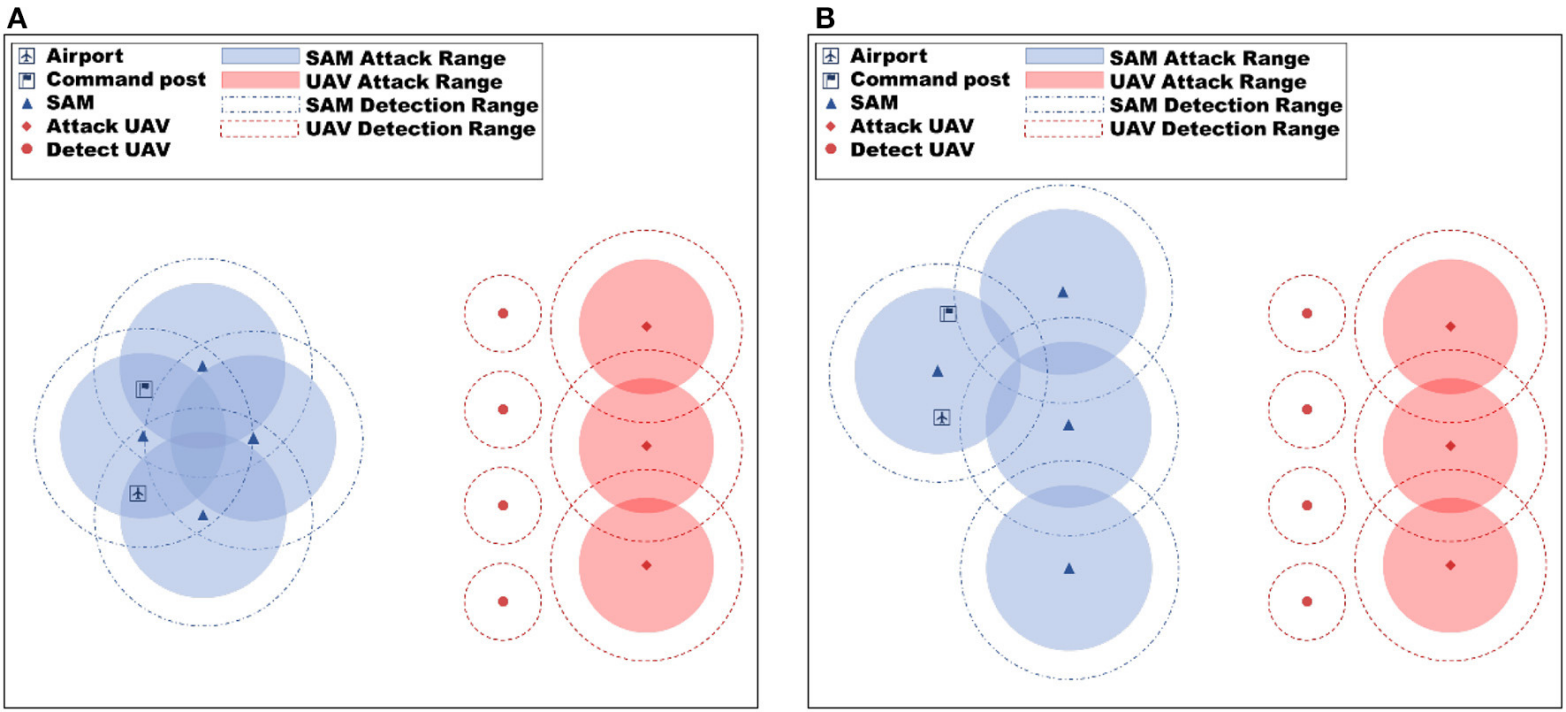
Training scenario experiment setup diagram, **(A)** is training scenario 1, and **(B)** is training scenario 2. Various training scenarios were employed to improve the generalization capacity of the proposed algorithm.

**Figure 5:**
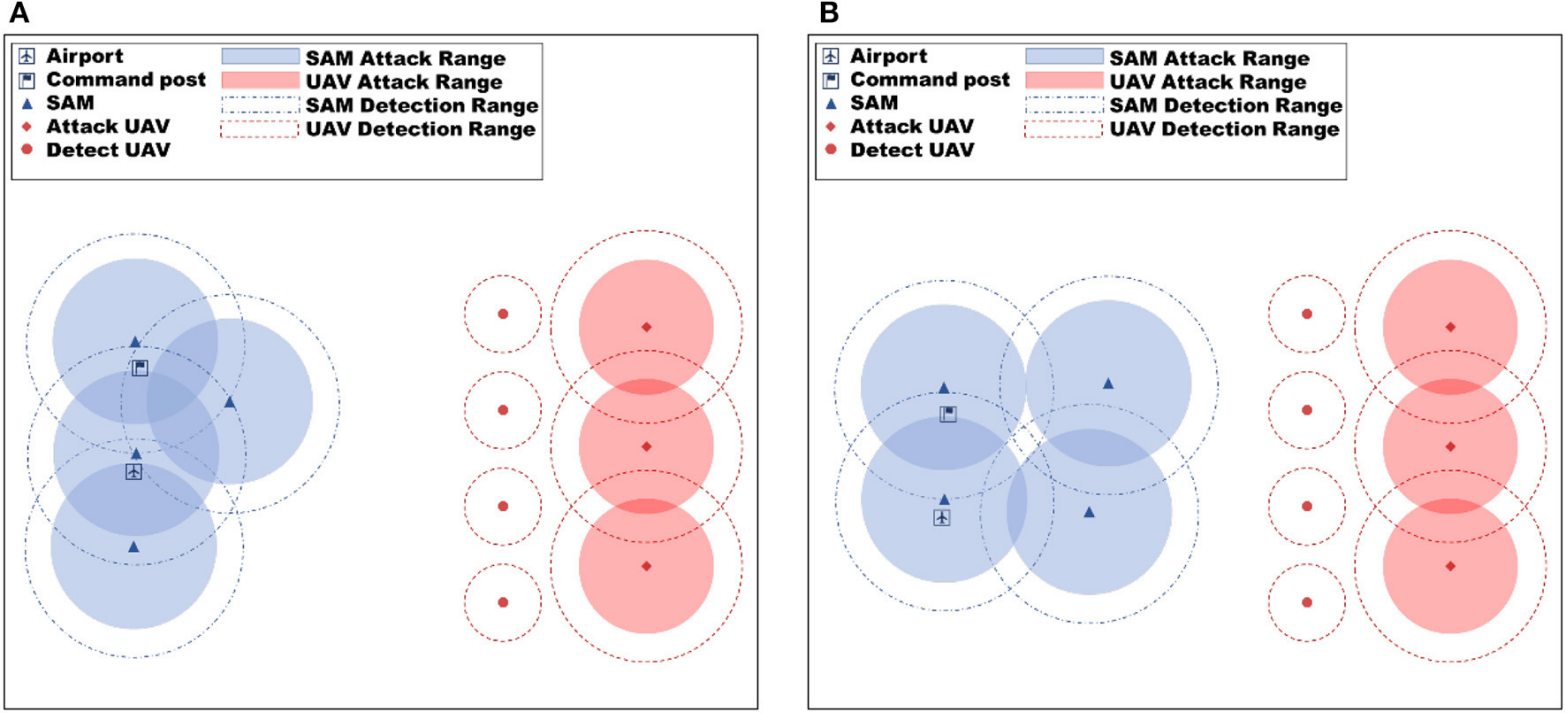
Test scenario experiment setup diagram, **(A)** is test scenario 1 and **(B)** is test scenario 2. Various test scenarios were employed to evaluate the generalization capacity of the proposed algorithm.

To assess the efficacy of the proposed method, the algorithm was validated in two simulation scenarios (as depicted in Figure 4 for training scenarios and Figure 5 for test scenarios) and compared against the MADDPG algorithm. The simulation scenarios are designed based on the force settings and battlefield environment assumptions described in Section 3. The primary focus of the evaluation is on the improved MAML method and the Reward-TD prioritized experience replay method proposed in this paper.

The simulation scenario consists of four red reconnaissance UAVs and three attack UAVs, whose objective is to destroy the opponent's command post. During training, the position of the red UAVs is fixed at the beginning of each episode, while the positions of the opponent's command post and SAM are changed in the two training scenarios to enable meta-training of the neural network. The training hardware used for the experiments includes Intel Xeon E5-4655V4 CPU with eight cores, 512 GB RAM, and RTX3060 GPU with 12GB video memory. The proposed method is implemented using a standard fully connected multilayer perception (MLP) network with ReLU nonlinearities, consisting of three hidden layers. The size of the experimental environment is 240 km × 240 km, and the hyperparameters used in the experiments are shown in Table 3, with the settings referred to from Xu et al. (2021). During meta-training, the meta-training process lasts for 5 × 10^5^ episodes to allow for sufficient learning and optimization of the neural network.

**Table 3:** Hyperparameter setting for training process.

**Hyperparameter**	**Value**
Replay buffer size	10^5^
Batch size	1,024
minibatch size	32
Discount factor	0.95
Actor learning rate	0.0001
Critic learning rate	0.0005
Prioritized experience replay parameter	0.6
Exponential decay rate	0.9
Exponential decay rate	0.999
Small constant	10^−4^
Act noise	Uhlenbeck-Ornstein (UO)
Weight learning rate	0.001
Meta-learning rate	0.001
Coefficient of the importance of the experience	0.4
Priority importance parameter	1
Sampling threshold	10
Soft update parameter	0.01
Active function	ReLU

### 5.2. Experiment result

This section aims to evaluate the meta-learning and cold-start capability of the proposed MW-MADDPG algorithm in new task environments, as well as its generalization, convergence speed, and robustness compared to existing algorithms. Additionally, the performance of the proposed Reward-TD prioritized experience replay method with the forgetting mechanism is evaluated and compared to conventional methods.

#### 5.2.1. Cross-task performance comparison

**Figure 6:**
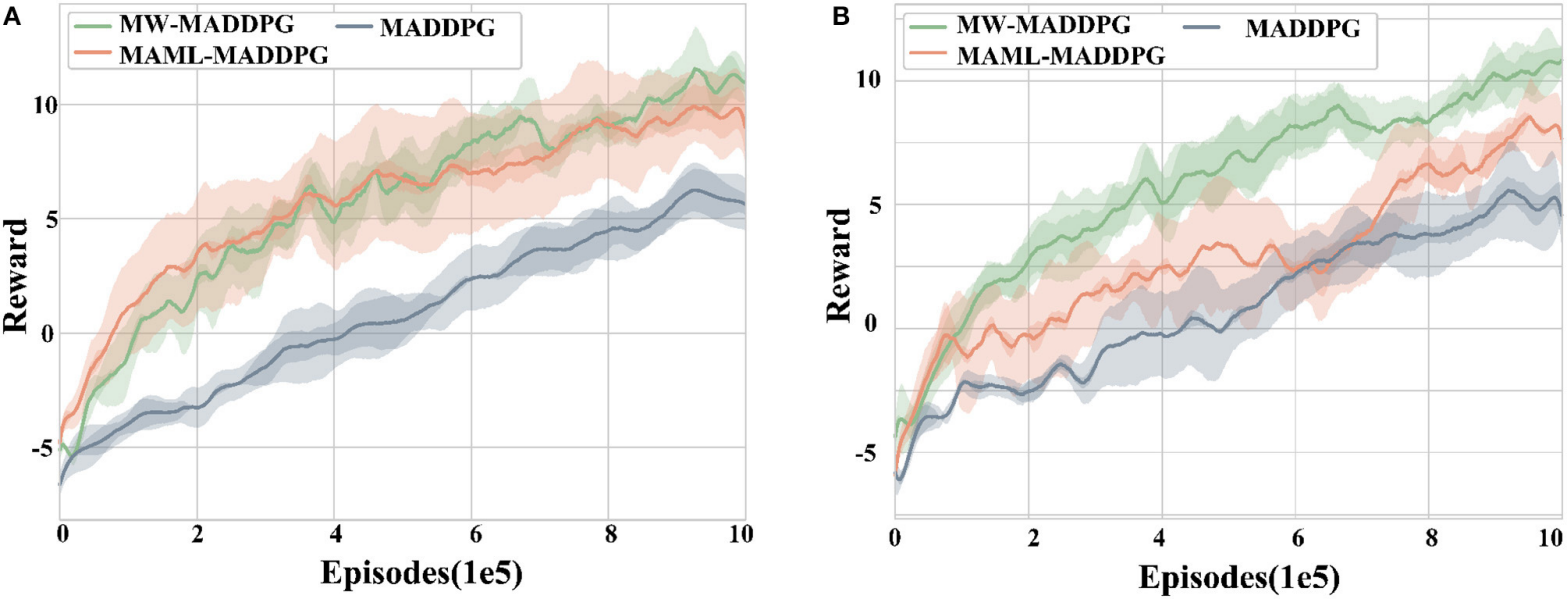
Test scenario experiment setup diagram, **(A)** is reward curve of test scenario 1 and **(B)** is reward curve of test scenario 2. Various test scenarios were employed to evaluate the generalization capacity of the proposed algorithm.

The performance of the three algorithms (MW-MADDPG, MAML-MADDPG, and MADDPG) is evaluated using the reward value as the evaluation index across five random seeds in the two scenarios, as shown in Figure 6. The results demonstrate that the MW-MADDPG and MAML-MADDPG algorithms with meta-learning outperform the MADDPG algorithm without meta-learning in both scenarios from the beginning episodes. Spechis indicates that the use of metaifically, the average reward for the MW-MADDPG method is −1.39, for the MAML-MADDPG method is −1.59, while the MADDPG method is −4.93 in scenario 1. In scenario 2, the average reward for the MW-MADDPG method is −2.25, for the MAML-MADDPG method is −2.18, and for the MADDPG method is −4.05.

Moreover, the initial performance of both methods employing meta-learning is significantly better than that of the MAML algorithm without meta-learning (*p* < 0.05). This indicates that the use of meta-learning methods can effectively improve the initial performance of the agent in this task.

In contrast, there is no significant difference between the initial performance of the MW-MADDPG method and the MAML-MADDPG method, indicating that the improvement in the initial performance of the proposed method in this paper is not statistically significant compared to existing reinforcement learning methods.

However, in terms of expected performance, the MW-MADDPG algorithm significantly outperforms the other two algorithms in terms of rewards when convergence is reached (*p* < 0.05). This suggests that the MW-MADDPG method proposed in this paper is capable of learning better strategies for the task at hand.

Regarding convergence rate, the MW-MADDPG algorithm reaches convergence at around 6 × 10^5^ episodes, while the MAML-MADDPG algorithm takes around 8.5 × 10^5^ episodes, and the MADDPG algorithm takes around 9 × 10^5^ episodes to converge for both scenarios. This indicates that the MW-MADDPG method proposed in this paper can converge quickly in a new task environment and alleviate the cold-start problem, showcasing an advantage over existing methods.

**Figure 7:**
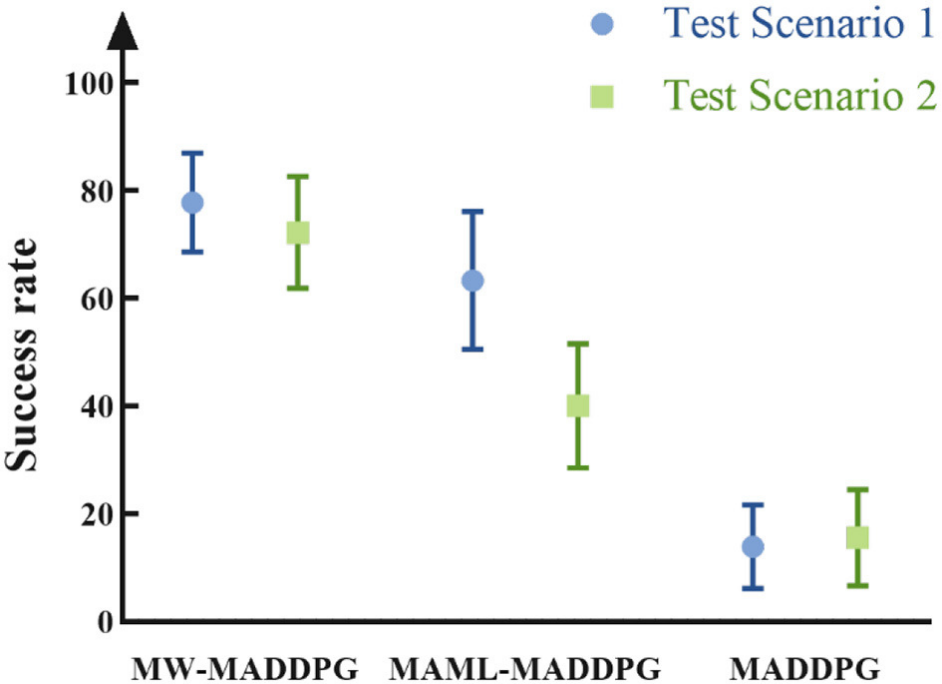
Red side task execution success rate. In various test scenarios, the proposed method exhibits a higher winning rate compared to both the traditional meta-learning method and the non-meta-learning method.

Figure 7 depicts the success rate of task execution in red, and it is evident that the MW-MADDPG method achieves a success rate of 77.71 and 72.21% in the two scenarios, respectively, which is significantly higher than the success rate of the other two methods (*p* < 0.05). These results indicate that the proposed method can effectively improve the performance of the agent under new tasks. Additionally, the variance of the MW-MADDPG method is smaller than that of the MAML-MADDPG method, indicating that the stability of the proposed method is better than that of the traditional meta-learning method.

Overall, the experiments demonstrate that the MW-MADDPG algorithm proposed in this paper can effectively learn the features of similar tasks, and learn from historical experience to obtain more effective strategies. The proposed method exhibits better initial performance, faster learning rate, better-expected performance, higher task success rate, and improved strategy stability in terms of reward and task execution success rate.

#### 5.2.2. Reward-TD and FIFO performance

This section aims to verify the effectiveness of the proposed Reward-TD prioritized experience replay method and forgetting mechanism. Two sets of experiments are designed to apply the above experience replay mechanism to the MADDPG algorithm in training scenario 1 and training scenario 2, respectively. The reward curves obtained by the agent are analyzed across five random seeds to evaluate the performance of the proposed method.

**Figure 8:**
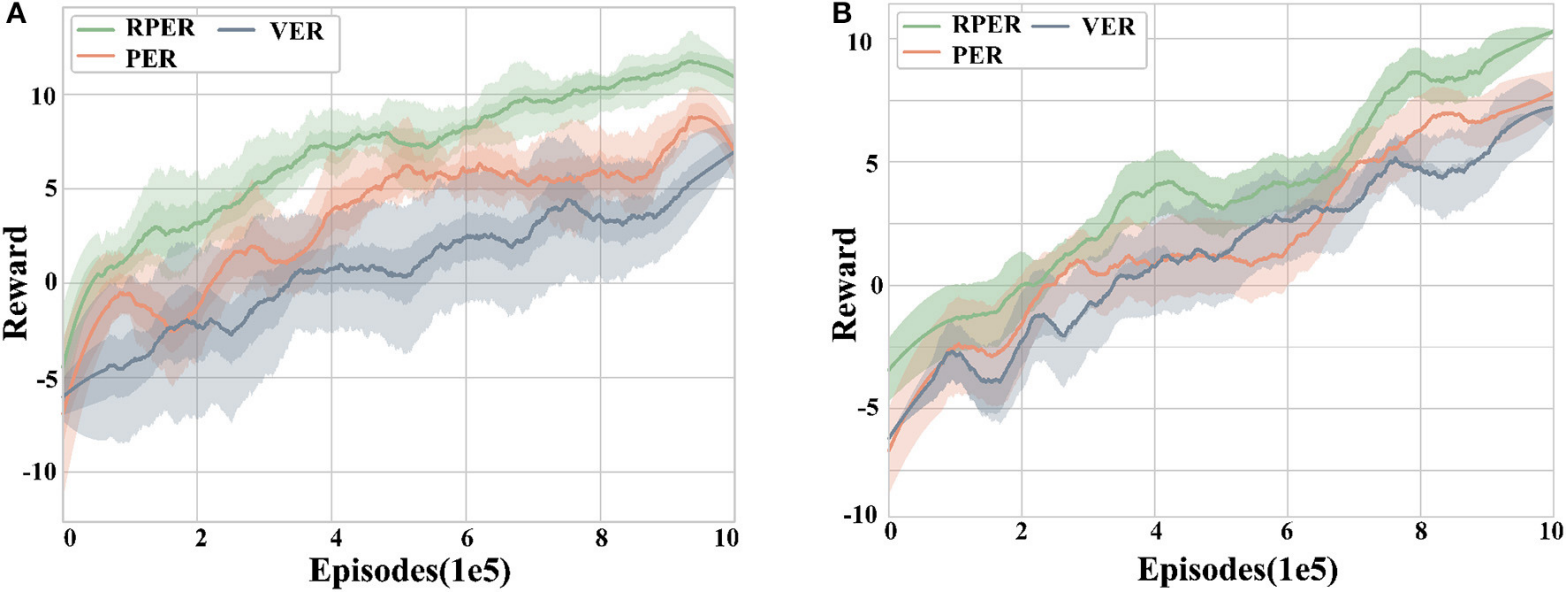
Reward curves for different experience replay methods. **(A)** Training scenario 1. **(B)** Training scenario 2. The RPER method outperforms the other two experience replay methods in terms of final reward, robustness, and algorithm convergence speed.

Figure 8 illustrates the reward curves of different experience replay methods in scenario 1 and scenario 2, with RPER representing the Reward-TD prioritized experience replay method, PER indicating the use of TD-error prioritized experience replay method, and VER standing for the random experience replay method. It can be observed that the final rewards obtained by using the RPER mechanism are significantly better than the other two methods (p < 0.05), indicating that the RPER mechanism can effectively improve the final reward level. In contrast, the difference between the final rewards of the PER and VER methods is not significant, suggesting that the TD-error-based preferred experience replay method has little effect on the final reward.

Regarding robustness, the RPER mechanism outperforms the PER mechanism, while the PER mechanism outperforms the VER mechanism. This indicates that the prioritized experience replay mechanism is better than the random uniform experience replay mechanism, and the Reward-TD based experience prioritization is better than the TD-error based experience prioritization.

In terms of convergence speed, the RPER algorithm achieves convergence significantly faster than the PER and VER algorithms. Specifically, RPER reaches convergence at around 8 × 10^5^ episodes in both scenarios, while PER and VER reach convergence only after around 9 × 10^5^ episodes. These results demonstrate that the RPER mechanism helps to improve the convergence speed of the algorithm, while PER and VER have no significant impact on the convergence speed.

**Figure 9:**
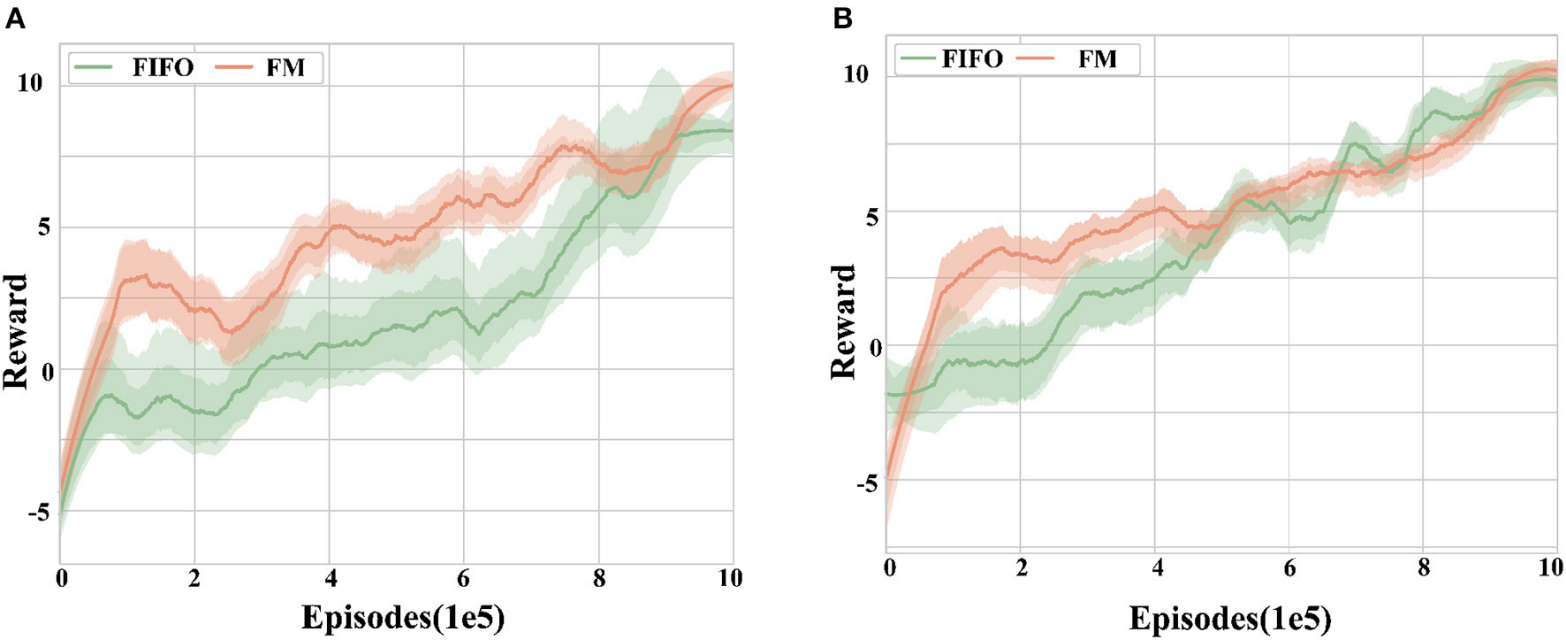
Reward curves for different experience retention methods. **(A)** Training scenario 1. **(B)** Training scenario 2. The forgetting mechanism shows better convergence speed and robustness compared to the first-in-first-out mechanism in different training tasks, but the difference in the final reward is not significant.

Figure 9 illustrates the graphs of different experience retention methods reward, comparing the effects of the forgetting mechanism (FM) and the first-in-first-out mechanism (FIFO) while using RPER and the MADDPG algorithm. From Figure 9, it can be observed that the training speed using the forgetting mechanism is significantly better than the FIFO mechanism in terms of convergence speed (*p* < 0.05). This suggests that the forgetting mechanism proposed in this paper can effectively retain experience fragments that are beneficial to the agent and improve the training speed.

In terms of robustness, the FM mechanism exhibits fewer curve fluctuations and a smaller range of error bands compared to the FIFO mechanism, as seen from the curve fluctuations and error band shading in the figure. The data show that the variance is reduced by 27.35% using FM compared to FIFO, indicating that FM can improve the algorithm's robustness during training.

Notably, there is no significant difference between the final rewards of the two experience retention mechanisms, suggesting that the use of different experience retention mechanisms has no significant effect on the final training effect.

Table 4 compares the proposed method with the original MADDPG method in terms of task success rate, strategic location ruin number, and other metrics to evaluate their advantages and disadvantages. The table shows that the proposed method outperforms the MADDPG method on both training and testing tasks. Specifically, the MW-MADDPG method exhibits significantly better attack UAV survival than detect UAV survival on testing tasks, indicating that it can learn an efficient strategy for attacking UAVs. These results suggest that the MW-MADDPG method proposed in this paper can effectively learn the common knowledge among tasks from training tasks and apply it to test scenarios, showcasing better cross-task capability.

**Table 4:** Algorithm performance comparison.

**Method**	**MADDPG**				**MW-MADDPG**			
**Scenario**	**Training scenario 1**	**Training scenario 2**	**Test scenario 1**	**Test scenario 2**	**Training scenario 1**	**Training scenario 2**	**Test scenario 1**	**Test scenario 2**
Reward	11.31 ± 1.31	11.52 ± 1.29	5.03 ± 1.88	5.12 ± 2.13	11.62 ± 1.14	11.58 ± 1.26	10.83 ± 1.53	10.48 ± 1.45
Mission success Rate	80.74 ± 7.84	82.47 ± 7.93	13.88 ± 7.76	15.55 ± 8.94	81.39 ± 6.49	79.85 ± 7.39	78.76 ± 7.94	75.48 ± 8.93
Strategic location Ruin number	0.93 ± 0.44	0.91 ± 0.36	0.12 ± 0.09	0.13 ± 0.08	0.85 ± 0.37	0.87 ± 0.31	0.81 ± 0.54	0.79 ± 0.66
Detect UAV Survival number	0.83 ± 0.53	0.88 ± 0.48	0.21 ± 0.13	0.19 ± 0.11	0.91 ± 0.47	0.83 ± 0.53	0.63 ± 0.37	0.71 ± 0.41
Attack UAV Survival number	1.66 ± 0.44	1.71 ± 0.39	0.37 ± 0.18	0.41 ± 0.21	1.74 ± 0.36	1.69 ± 0.41	1.38 ± 0.47	1.41 ± 0.51

Furthermore, the proposed Reward-TD prioritized experience replay method with the forgetting mechanism can improve the algorithm's robustness, exhibiting less variance and greater robustness for the MW-MADDPG method.

## 6. Conclusion

In summary, this paper proposes the MW-MADDPG algorithm for the cross-task heterogeneous UAV swarm cooperative decision-making problem. The proposed algorithm includes the improved MAML meta-learning method and the Reward-TD priority reward replay method with a forgetting mechanism, enabling cross-task intelligent UAV decision-making based on the MADDPG algorithm and achieving the expected goals. Experimental results demonstrate that the proposed methods can achieve better task success rates, robustness, and rewards compared to traditional methods, while also exhibiting better generalization performance, overcoming the cold start problem in traditional methods. The proposed algorithm has the potential to be extended to larger-scale scenarios and provide a solution to the cross-task heterogeneous UAV swarm surprise defense problem.

In the future, further research can be done by introducing meta-learning methods into intelligent decision-making in air defense systems to enable self-play between UAV penetration and air defense systems. Additionally, combining transfer learning with meta-learning may improve generalization performance. Furthermore, we prepare to build a high-fidelity battlefield environment that can provide a more accurate simulation of the battle process and enable more realistic testing of the proposed algorithms.

## Data availability statement

The raw data supporting the conclusions of this article will be made available by the authors, without undue reservation.

## Author contributions

Conceptualization: MZ and QF. Data curation: XG. Funding acquisition and writing—review and editing: GW. Investigation: MZ, XG, YC, and XL. Methodology and writing—original draft: MZ. Software: TL. Supervision: GW and QF. Validation: QF and YC. Visualization: MZ, TL, and XL. All authors contributed to the article and approved the submitted version.
